# ITS-90 Density of Water Formulation for Volumetric Standards Calibration

**DOI:** 10.6028/jres.097.013

**Published:** 1992

**Authors:** Frank E. Jones, Georgia L. Harris

**Affiliations:** 32 Orchard Way South, Potomac, MD 20854; National Institute of Standards and Technology, Gaithersburg, MD 20899

**Keywords:** air-saturated water, calibration, density of water, isothermal compressibility, ITS-90, volumetric standards

## Abstract

A new formulation of the density of air-saturated water as a function of temperature on the 1990 International Temperature Scale (ITS-90) is presented. Also, a new equation for calculating isothermal compressibility as a function of temperature on ITS-90 was developed. The equations are to be used to calculate the density of water, in the temperature range 5 to 40 °C on ITS-90, used in the gravimetric determination of the volume of volumetric standards.

## 1. Introduction

In the gravimetric determination of the volume (calibration) of volumetric standards, water is used as the calibrating fluid. The volume is calculated from the mass and density of the water. In many quarters, the formulation of Wagenbreth and Blanke [1] is used to calculate the density of water. In this paper, a new formulation of the density of water (based primarily on the work of Kell [2]) as a function of temperature on the 1990 International Temperature Scale is presented.

## 2. Kelt’s Formulations

### 2.1 Density of Water

In 1975, Kell [2] published a new formulation for the density of *air-free* water at a pressure of 101.325 kPa (1 atmosphere) valid from 0 to 150 °C “that is in improved agreement with most data sets.” The Kell formulation is

$$\begin{array}{l} \rho \left(\mathrm{kg}\;m^{-3}\right)=(999.83952+16.945176\;t\\ -7.9870401\times 10^{-3}t^{2}-46.170461\times 10^{-6}t^{3}\\ +105.56302\times 10^{-9}t^{4}-280.54253\times 10^{-12}t^{5})\\ \;/(1+16.897850\times 10^{-3}t, \end{array}$$ (1)

where *t* is temperature in °C on the 1968 International Practical Temperature Scale (IPTS-68).

### 2.2 Isothermal Compressibility

Kell also developed equations for calculation of the isothermal compressibility, *κ_T_*, of air-free water [2]. In the temperature range 0 to 100 °C on IPTS-68, the equation can be expressed as

$$\begin{array}{l} \kappa_{T}=(50.88496\times 10^{-8}+6.163813\times 10^{-9}t\\ +1.459187\times 10^{-11}t^{2}+20.08438\times 10^{-14}t^{3}\\ -58.47727\times 10^{-17}t^{4}+410.4110\times 10^{-20}t^{5})\\ \;/\left(1+19.67348\times 10^{-3}t\right), \end{array}$$ (2)

where *κ_T_* is isothermal compressibility in (kPa)^−1^.

## 3. New Formulations

### 3.1 Density of Air-Free Water

In the present work, the Kell calculated values of *ρ* were fitted over the temperature range 5 to 40 °C on the new 1990 International Temperature Scale (ITS-90) [3] to an equation quartic in temperature. The equation is

$$\begin{array}{l} \rho \left(\mathrm{kg}\;m^{-3}\right)=999.85308+6.32693\times 10^{-2}t\\ \;-8.523829\times 10^{-3}t^{2}+6.943248\times 10^{-5}t^{3}\\ \;-3.821216\times 10^{-7}t^{4}. \end{array}$$ (3)

In contrast with the Kell equation, a term in *t^5^* is not necessary due at least in part to the fact that the 0 to 4 °C region, in which *ρ* increases with increasing temperature, has been excluded. Equation (3) applies to *air-free* water.

Values of the density of air-free water were calculated for temperatures (ITS-90, *t*_90_) between 4.999 and 39.990 °C using Eq. (3) and compared with corresponding Kell values. The estimate of the standard deviation (SD) of the difference was 0.00034 kg m^−3^. The ratio of SD to the mean value of density was 3.4 × 10^−7^, which is negligible.

### 3.2 Conversion of IPTS-68 to ITS-90

A very simple equation relating ITS-90 temperature, *t*_90_ to IPTS-68 temperature, *t*_68_, has been used in the present work to generate values of *t*_90_ for the development of Eq. (3). The equation for the temperature range 0 to 40 °C is

$$t_{90}=0.0002+0.99975\;t_{68}.$$ (4a)

In the temperature range 0 to 100 °C the equation is

$$t_{90}=0.0005+0.9997333\;t_{68}.$$ (4b)

### 3.3 Change in Density of Water with Air Saturation

Bignell [4] measured the change in the density of water with air saturation for 80 points in the range of 4 to 20 °C. He fitted the points to develop the equation

Δρ=−0.004612+0.000106t, (5)

where Δ*ρ* is in kg m^−3^. There is no need to adjust for temperature scale. Bignell concluded that “there is probably not much need to extend the work to higher temperatures because the effect diminishes and the accuracy of density metrology at these temperatures would not warrant a more accurately known correction.”

### 3.4 Density of Air-Saturated Water on ITS-90

Equation (5) was added to Eq. (3) to produce an equation to be used to calculate the density, *ρ*_as_, of *air-saturated* water in the temperature range 5 to 40 °C on ITS-90:

$$\begin{array}{l} \rho_{\mathrm{as}}=999.84847+6.337563\times 10^{-2}\;t\\ \;-8.523829\times 10^{-3}t^{2}+6.943248\times 10^{-5}\;t^{3}\\ \;-3.821216\times 10^{-7}\;t^{4} \end{array}$$ (6)

The uncertainty in the density of air-saturated water for an uncertainty in temperature of 1 °C is approximately 210 ppm or 0.21 kg m^−3^ at 20 °C.

### 3.5 Isothermal Compressibility

The thermal compressibility data used by Kell have been fitted against temperature on ITS-90 for the temperature range 5 to 40 °C. The resulting equation is

$$\begin{array}{l} \kappa_{T}=50.83101\times 10^{-8}-3.68293\times 10^{-9}\;t\\ \;+7.263725\times 10^{-11}\;t^{2}-6.597702\times 10^{-13}\;t^{3}\\ \;+2.87767\times 10^{-15}\;t^{4}, \end{array}$$ (7)

where *κ_T_* is thermal compressibility in (kPa)^−1^.

The estimate of standard deviation (SD) of the residual, calculated *κ_T_*–data *κ_T_*, is 2.1 × l0^−11^ (kPa)^−1^; the ratio of SD to the midrange value of *κ_T_* is 4.6 × l0^−5^, which is negligible for present purposes. It is not necessary to make a correction to *κ_T_* for air saturation.

The value of the isothermal compressibility of water is approximately 46.5 parts per million (ppm)/atmosphere at 20 °C. At locations where the atmospheric pressure is significantly different from 1 atmosphere (101.325 kPa), a correction for compressibility calculated using Eq. (7) should be made. For example, at Boulder, CO, the correction for compressibility is approximately −8 ppm at 20 °C.

### 3.6 Compressibility-Corrected Water Density Equation

The expression for the density of air-saturated water, *ρ*_asc_, at an ambient pressure of *P* kPa is

$$\rho_{\mathrm{asc}}=\rho_{\mathrm{as}}\left[1+\kappa_{T}\left(P-101.325\right)\right],$$ (8)

where *ρ*_as_ is calculated using Eq. (6) and *κ_T_* is calculated using Eq. (7).

## 4. Tables

Table 1 is a tabulation of values of the density of air-saturated water using Eq. (6). Table 2 is a tabulation of the values of the density of air-free water calculated using Eq. (3). Table 3 is a tabulation of values of air-free water calculated using the formulation of Wagenbreth and Blanke [1], this table has been included in this paper for purposes of comparison.

The units for water density in these tables are g/cm^3^, as a convenience to those who routinely use these units.

## 5. Summary

Equation (3) can be used to calculate the density of *air-free* water in the temperature range of 5 to 40 °C in ITS-90 at one atmosphere.

Equation (6) can be used to calculate the density of *air-saturated* water in the same temperature range at one atmosphere.

Equation (8) can be used to calculate the density of air-saturated water in the same temperature range at an ambient pressure of *P* kPa.

The use of Eq. (6) for air-saturated water, and Eq. (8) where appropriate, is recommended for calculation of water density.

## Figures and Tables

**Table 1:** Density of *air-saturated* water (g/cm^3^)from Eq. (6) using Kell [2] data

*t*(°C)	0.0	0.1	0.2	0.3	0.4	0.5	0.6	0.7	0.8	0.9
5	0.999961	0.999959	0.999957	0.999955	0.999953	0.999950	0.999948	0.999945	0.999942	0.999939
6	0.999936	0.999933	0.999930	0.999926	0.999922	0.999919	0.999915	0.999911	0.999906	0.999902
7	0.999897	0.999893	0.999888	0.999883	0.999878	0.999872	0.999867	0.999861	0.999856	0.999850
8	0.999844	0.999838	0.999832	0.999825	0.999819	0.999812	0.999805	0.999798	0.999791	0.999784
9	0.999777	0.999769	0.999761	0.999754	0.999746	0.999738	0.999730	0.999721	0.999713	0.999704
10	0.999695	0.999687	0.999678	0.999669	0.999659	0.999650	0.999640	0.999631	0.999621	0.999611
11	0.999601	0.999591	0.999581	0.999570	0.999560	0.999549	0.999538	0.999527	0.999516	0.999505
12	0.999494	0.999482	0.999471	0.999459	0.999447	0.999435	0.999423	0.999411	0.999398	0.999386
13	0.999373	0.999361	0.999348	0.999335	0.999322	0.999309	0.999295	0.999282	0.999268	0.999255
14	0.999241	0.999227	0.999213	0.999199	0.999184	0.999170	0.999156	0.999141	0.999126	0.999111
15	0.999096	0.999081	0.999066	0.999051	0.999035	0.999019	0.999004	0.998988	0.998972	0.998956
16	0.998940	0.998923	0.998907	0.998891	0.998874	0.998857	0.998840	0.998823	0.998806	0.998789
17	0.998772	0.998754	0.998737	0.998719	0.998701	0.998683	0.998665	0.998647	0.998629	0.998611
18	0.998592	0.998574	0.998555	0.998536	0.998517	0.998499	0.998479	0.998460	0.998441	0.998421
19	0.998402	0.998382	0.998363	0.998343	0.998323	0.998303	0.998283	0.998262	0.998242	0.998221
20	0.998201	0.998180	0.998159	0.998138	0.998117	0.998096	0.998075	0.998054	0.998032	0.998011
21	0.997989	0.997967	0.997945	0.997924	0.997901	0.997879	0.997857	0.997835	0.997812	0.997790
22	0.997767	0.997744	0.997721	0.997698	0.997675	0.997652	0.997629	0.997606	0.997582	0.997559
23	0.997535	0.997511	0.997487	0.997463	0.997439	0.997415	0.997391	0.997366	0.997342	0.997317
24	0.997293	0.997268	0.997243	0.997218	0.997193	0.997168	0.997143	0.997118	0.997092	0.997067
25	0.997041	0.997015	0.996990	0.996964	0.996938	0.996912	0.996885	0.996859	0.996833	0.996806
26	0.996780	0.996753	0.996726	0.996700	0.996673	0.996646	0.996619	0.996591	0.996564	0.996537
27	0.996509	0.996482	0.996454	0.996426	0.996399	0.996371	0.996343	0.996314	0.996286	0.996258
28	0.996230	0.996201	0.996173	0.996144	0.996115	0.996086	0.996057	0.996028	0.995999	0.995970
29	0.995941	0.995912	0.995882	0.995853	0.995823	0.995793	0.995764	0.995734	0.995704	0.995674
30	0.995643	0.995613	0.995583	0.995553	0.995522	0.995491	0.995461	0.995430	0.995399	0.995368
31	0.995337	0.995306	0.995275	0.995244	0.995212	0.995181	0.995149	0.995118	0.995086	0.995054
32	0.995023	0.994991	0.994959	0.994927	0.994894	0.994862	0.994830	0.994797	0.994765	0.994732
33	0.994699	0.994667	0.994634	0.994601	0.994568	0.994535	0.994502	0.994468	0.994435	0.994402
34	0.994368	0.994334	0.994301	0.994267	0.994233	0.994199	0.994165	0.994131	0.994097	0.994063
35	0.994028	0.993994	0.993960	0.993925	0.993890	0.993856	0.993821	0.993786	0.993751	0.993716
36	0.993681	0.993646	0.993610	0.993575	0.993539	0.993504	0.993468	0.993433	0.993397	0.993361
37	0.993325	0.993289	0.993253	0.993217	0.993181	0.993144	0.993108	0.993071	0.993035	0.992998
38	0.992962	0.992925	0.992888	0.992851	0.992814	0.992777	0.992740	0.992702	0.992665	0.992628
39	0.992590	0.992553	0.992515	0.992477	0.992439	0.992401	0.992363	0.992325	0.992287	0.992249

**Table 2:** Density of *air-free* water (g/cm^3^) from Eq. (3) using Kell [2] data

*t*(°C)	0.0	0.1	0.2	0.3	0.4	0.5	0.6	0.7	0.8	0.9
5	0.999965	0.999963	0.999961	0.999959	0.999957	0.999954	0.999952	0.999949	0.999946	0.999943
6	0.999940	0.999937	0.999934	0.999930	0.999926	0.999923	0.999919	0.999914	0.999910	0.999906
7	0.999901	0.999896	0.999892	0.999887	0.999881	0.999876	0.999871	0.999865	0.999860	0.999854
8	0.999848	0.999842	0.999835	0.999829	0.999822	0.999816	0.999809	0.999802	0.999795	0.999788
9	0.999780	0.999773	0.999765	0.999757	0.999749	0.999741	0.999733	0.999725	0.999716	0.999708
10	0.999699	0.999690	0.999681	0.999672	0.999663	0.999653	0.999644	0.999634	0.999624	0.999615
11	0.999604	0.999594	0.999584	0.999574	0.999563	0.999552	0.999541	0.999531	0.999519	0.999508
12	0.999497	0.999485	0.999474	0.999462	0.999450	0.999438	0.999426	0.999414	0.999402	0.999389
13	0.999377	0.999364	0.999351	0.999338	0.999325	0.999312	0.999299	0.999285	0.999272	0.999258
14	0.999244	0.999230	0.999216	0.999202	0.999188	0.999173	0.999159	0.999144	0.999129	0.999114
15	0.999099	0.999084	0.999069	0.999053	0.999038	0.999022	0.999007	0.998991	0.998975	0.998959
16	0.998943	0.998926	0.998910	0.998893	0.998877	0.998860	0.998843	0.998826	0.998809	0.998792
17	0.998774	0.998757	0.998739	0.998722	0.998704	0.998686	0.998668	0.998650	0.998632	0.998613
18	0.998595	0.998576	0.998558	0.998539	0.998520	0.998501	0.998482	0.998463	0.998444	0.998424
19	0.998405	0.998385	0.998365	0.998345	0.998325	0.998305	0.998285	0.998265	0.998244	0.998224
20	0.998203	0.998183	0.998162	0.998141	0.998120	0.998099	0.998077	0.998056	0.998035	0.998013
21	0.997991	0.997970	0.997948	0.997926	0.997904	0.997882	0.997859	0.997837	0.997815	0.997792
22	0.997769	0.997746	0.997724	0.997701	0.997678	0.997654	0.997631	0.997608	0.997584	0.997561
23	0.997537	0.997513	0.997489	0.997465	0.997441	0.997417	0.997393	0.997369	0.997344	0.997320
24	0.997295	0.997270	0.997245	0.997220	0.997195	0.997170	0.997145	0.997120	0.997094	0.997069
25	0.997043	0.997017	0.996992	0.996966	0.996940	0.996914	0.996887	0.996861	0.996835	0.996808
26	0.996782	0.996755	0.996728	0.996701	0.996675	0.996648	0.996620	0.996593	0.996566	0.996539
27	0.996511	0.996483	0.996456	0.996428	0.996400	0.996372	0.996344	0.996316	0.996288	0.996260
28	0.996231	0.996203	0.996174	0.996146	0.996117	0.996088	0.996059	0.996030	0.996001	0.995972
29	0.995942	0.995913	0.995884	0.995854	0.995824	0.995795	0.995765	0.995735	0.995705	0.995675
30	0.995645	0.995615	0.995584	0.995554	0.995523	0.995493	0.995462	0.995431	0.995401	0.995370
31	0.995339	0.995307	0.995276	0.995245	0.995214	0.995182	0.995151	0.995119	0.995087	0.995056
32	0.995024	0.994992	0.994960	0.994928	0.994895	0.994863	0.994831	0.994798	0.994766	0.994733
33	0.994701	0.994668	0.994635	0.994602	0.994569	0.994536	0.994503	0.994469	0.994436	0.994403
34	0.994369	0.994335	0.994302	0.994268	0.994234	0.994200	0.994166	0.994132	0.994098	0.994064
35	0.994029	0.993995	0.993960	0.993926	0.993891	0.993856	0.993822	0.993787	0.993752	0.993717
36	0.993682	0.993646	0.993611	0.993576	0.993540	0.993505	0.993469	0.993433	0.993398	0.993362
37	0.993326	0.993290	0.993254	0.993217	0.993181	0.993145	0.993108	0.993072	0.993035	0.992999
38	0.992962	0.992925	0.992888	0.992851	0.992814	0.992777	0.992740	0.992703	0.992665	0.992628
39	0.992590	0.992553	0.992515	0.992478	0.992440	0.992402	0.992364	0.992326	0.992288	0.992249

**Table 3:** Density of *air-free* water (g/cm^3^) from formulation of Wagenbreth and Blanke [1]

*t*(°C)	0.0	0.1	0.2	0.3	0.4	0.5	0.6	0.7	0.8	0.9
5	0.999964	0.999962	0.999960	0.999958	0.999956	0.999954	0.999951	0.999949	0.999946	0.999943
6	0.999940	0.999937	0.999933	0.999930	0.999926	0.999922	0.999918	0.999914	0.999910	0.999906
7	0.999901	0.999896	0.999892	0.999887	0.999881	0.999876	0.999871	0.999865	0.999860	0.999854
8	0.999848	0.999842	0.999835	0.999829	0.999822	0.999816	0.999809	0.999802	0.999795	0.999787
9	0.999780	0.999773	0.999765	0.999757	0.999749	0.999741	0.999733	0.999725	0.999716	0.999707
10	0.999699	0.999690	0.999681	0.999672	0.999662	0.999653	0.999643	0.999634	0.999624	0.999614
11	0.999604	0.999594	0.999583	0.999573	0.999562	0.999552	0.999541	0.999530	0.999519	0.999507
12	0.999496	0.999485	0.999473	0.999461	0.999449	0.999437	0.999425	0.999413	0.999401	0.999388
13	0.999376	0.999363	0.999350	0.999337	0.999324	0.999311	0.999297	0.999284	0.999270	0.999256
14	0.999243	0.999229	0.999215	0.999200	0.999186	0.999172	0.999157	0.999142	0.999128	0.999113
15	0.999098	0.999083	0.999067	0.999052	0.999036	0.999021	0.999005	0.998989	0.998973	0.998957
16	0.998941	0.998925	0.998908	0.998892	0.998875	0.998858	0.998841	0.998824	0.998807	0.998790
17	0.998773	0.998755	0.998738	0.998720	0.998702	0.998684	0.998666	0.998648	0.998630	0.998612
18	0.998593	0.998575	0.998556	0.998537	0.998519	0.998500	0.998480	0.998461	0.998442	0.998422
19	0.998403	0.998383	0.998364	0.998344	0.998324	0.998304	0.998284	0.998263	0.998243	0.998222
20	0.998202	0.998181	0.998160	0.998139	0.998118	0.998097	0.998076	0.998055	0.998033	0.998012
21	0.997990	0.997968	0.997947	0.997925	0.997903	0.997881	0.997858	0.997836	0.997814	0.997791
22	0.997768	0.997746	0.997723	0.997700	0.997677	0.997654	0.997630	0.997607	0.997584	0.997560
23	0.997536	0.997513	0.997489	0.997465	0.997441	0.997417	0.997392	0.997368	0.997344	0.997319
24	0.997294	0.997270	0.997245	0.997220	0.997195	0.997170	0.997145	0.997119	0.997094	0.997068
25	0.997043	0.997017	0.996991	0.996966	0.996940	0.996913	0.996887	0.996861	0.996835	0.996808
26	0.996782	0.996755	0.996723	0.996702	0.996675	0.996648	0.996621	0.996593	0.996566	0.996539
27	0.996511	0.996484	0.996456	0.996428	0.996401	0.996373	0.996345	0.996316	0.996288	0.996260
28	0.996232	0.996203	0.996175	0.996146	0.996117	0.996088	0.996060	0.996031	0.996001	0.995972
29	0.996943	0.995914	0.995884	0.995855	0.995825	0.995795	0.995765	0.995736	0.995706	0.995676
30	0.995645	0.995615	0.995585	0.995554	0.995524	0.995493	0.995463	0.995432	0.995401	0.995370
31	0.995339	0.995308	0.995277	0.995246	0.995214	0.995183	0.995151	0.995120	0.995088	0.995056
32	0.995024	0.994992	0.994960	0.994928	0.994896	0.994864	0.994831	0.994799	0.994766	0.994734
33	0.994701	0.994668	0.994635	0.994602	0.994569	0.994536	0.994503	0.994470	0.994436	0.994403
34	0.994369	0.994336	0.994302	0.994268	0.994234	0.994201	0.994167	0.994132	0.994098	0.994064
35	0.994030	0.993995	0.993961	0.993926	0.993891	0.993857	0.993822	0.993787	0.993752	0.993717
36	0.993682	0.993647	0.993611	0.993576	0.993541	0.993505	0.993469	0.993434	0.993398	0.993362
37	0.993326	0.993290	0.993254	0.993218	0.993182	0.993146	0.993109	0.993073	0.993036	0.993000
38	0.992963	0.992926	0.992889	0.992852	0.992815	0.992778	0.992741	0.992704	0.992667	0.992629
39	0.992592	0.992554	0.992517	0.992479	0.992442	0.992404	0.992366	0.992328	0.992290	0.992252

## References

[1] Wagenbreth H, Blanke W (1971). PTB Mitt.

[2] Kell GS (1975). J Chera Eng Data.

[3] Mangura BW, Furukawa GT (1990). Natl Inst Stand Technol.

[4] Bignell N (1983). Metrologia.

